# Exposure to Court-Ordered Tobacco Industry Antismoking Advertisements Among US Adults

**DOI:** 10.1001/jamanetworkopen.2019.6935

**Published:** 2019-07-12

**Authors:** Onyema Greg Chido-Amajuoyi, Robert K. Yu, Israel Agaku, Sanjay Shete

**Affiliations:** 1 Department of Epidemiology, The University of Texas MD Anderson Cancer Center, Houston; 2 Department of Biostatistics, The University of Texas MD Anderson Cancer Center, Houston; 3 Department of Oral Health Policy and Epidemiology, Harvard School of Dental Medicine, Boston, Massachusetts; 4 Division of Cancer Prevention and Population Sciences, The University of Texas MD Anderson Cancer Center, Houston

## Abstract

**Importance:**

In 2006, US District Judge Gladys Kessler ordered tobacco companies to make corrective statements through paid advertisements informing the public of their deceptive practices. This landmark ruling and its subsequent execution represent the first time the tobacco industry sponsored a nationwide corrective advertising campaign against its own products.

**Objective:**

To assess the reach of the court-ordered antismoking advertisements within the US adult population, stratified by demographic characteristics and tobacco use.

**Design, Setting, and Participants:**

This nationally representative, population-based cross-sectional survey of US adults included respondents to the 2018 Health Information National Trends Survey 5, Cycle 2. Respondents were representatives of households selected by equal-probability sampling of the Marketing Systems Group database of addresses that included all nonvacant US residential addresses. Data collection was conducted from January to May 2018, and analysis took place from December 2018 to April 2019.

**Main Outcomes and Measures:**

Self-reported exposure to court-ordered antismoking advertisements.

**Results:**

The overall sample of 3484 respondents included 2054 women (weighted percentage, 50.8%), 1976 non-Hispanic white respondents (weighted percentage, 59.9%), 2952 respondents who lived in urban US areas (weighted percentage, 84.9%), and 450 current smokers (weighted percentage, 15.6%). Estimated exposure to court-ordered antismoking advertisements was 40.6% (95% CI, 37.5%-43.7%) among the full sample and 50.5% (95% CI, 41.4%-59.6%) among current smokers. Exposure was lowest among those aged 18 to 34 years (37.4%; 95% CI, 28.0%-46.8%), those with a high school education or less (34.5%; 95% CI, 29.3%-39.8%), and those with household annual income less than $35 000 (37.5%; 95% CI, 32.0%-42.9%). Among current smokers, Hispanic respondents had lower exposure rates (42.2%; 95% CI, 18.5%-65.9%) than non-Hispanic white respondents (51.7%; 95% CI, 40.4%-63.1%). As the advertising campaign’s duration increased, exposure rates increased. Individuals with a high school education or less had lower odds of antismoking advertisement exposure than those with college or postgraduate degrees (adjusted odds ratio, 0.67; 95% CI, 0.48-0.94). Current smokers had higher odds of exposure than never smokers (adjusted odds ratio, 1.81; 95% CI, 1.17-2.80). Among those exposed to antismoking advertisements, 70.5% saw multiple antismoking messages.

**Conclusions and Relevance:**

Approximately 1 of 2 smokers reported exposure to the federal court–ordered antismoking advertisements. However, exposure was relatively lower among several subgroups, including individuals aged 18 to 34 years, only one-third of whom reported exposure. Increasing the duration of antismoking advertisements as well as expanding their coverage to youth-oriented media may increase their potential public health impact.

## Key Points

**Question:**

What was the reach of federal court–ordered antismoking advertisements among the US adult population?

**Findings:**

In a cross-sectional survey of US adults, estimated exposure to federal court–ordered antismoking advertisements was 40.6%. Exposure rates were lowest among those aged 18 to 34 years (37.4%), those with a high school education or less (34.5%), those earning less than $35 000 per year (37.5%), and Hispanic current smoker respondents (42.2%).

**Meaning:**

Penetration of the tobacco industry–funded antismoking advertisements was suboptimal within the US population and in populations at greatest risk of tobacco use.

## Teaser

This cross-sectional study assesses the reach of court-ordered antismoking advertisements among the US adult population, stratified by demographic characteristics and tobacco use.

## Introduction

In 1999, amid mounting evidence of deliberate and concerted efforts by the tobacco industry to mislead the general public about the health risks of smoking, the US Department of Justice filed a lawsuit against the industry for violating the Racketeer Influenced and Corrupt Organizations Act.^1^ In 2006, US District Judge Gladys Kessler ruled in favor of the Department of Justice and instructed tobacco companies to issue corrective statements informing the public of their deceptive practices, which spanned several decades, regarding the following areas: (1) the adverse health effects of smoking; (2) the addictiveness of smoking and nicotine; (3) the lack of significant harm reduction from smoking low-tar, light, ultra-light, mild, and natural cigarettes; (4) the manipulation of cigarette design to boost nicotine delivery; and (5) the adverse health effects of exposure to secondhand smoke.^1^ Judge Kessler ordered tobacco companies to sponsor the dissemination of these corrective messages through paid advertisements in major newspapers, on television, retail point-of-sale displays, cigarette package onserts, and their corporate websites.^1^ Following several years of litigation and appeals,^2,3^ the biggest tobacco companies in the United States began sponsoring antismoking advertisements, which appeared for the first time on prime time television and in major newspapers across the country in November 2017.^3,4^ While the TV and newspaper advertising campaigns are now ended, court-ordered antismoking advertising via cigarette package onserts and tobacco company websites are ongoing, but point-of-sale displays remain subject to litigation.^5^

Antismoking mass media campaigns are an effective public health intervention,^6,7,8^ and Article 12 of the World Health Organization Framework Convention on Tobacco Control lists such campaigns as a measure that must be adopted by all countries to curb tobacco use.^9^ When disseminated effectively, corrective advertising has been shown to be effective at reversing misconceptions. Prior to the finalization of the corrective statements, a 2011 study examined the effectiveness of different versions proposed by the Department of Justice, the tobacco industry, interveners, and research study investigators^10^ and found that corrective statements were highly effective at reversing misconceptions about smoking. A 2010 study^11^ examined the corrective statements and consumer beliefs about smoking and found a positive association of these statements with antismoking beliefs.

To our knowledge, since the dissemination of the corrective advertising campaign, no assessment has been conducted to evaluate the penetration of these advertisements within the US population. Given the tobacco industry’s history of deceptiveness as well as the skepticism generated by an industry-sponsored public health campaign (albeit a federal court–ordered campaign), an assessment of the population-level penetration of this antismoking advertising campaign is crucial.

Therefore, the purpose of this study was to assess the reach of these antismoking messages within the general population and at-risk groups, particularly current smokers. We examined rates and correlates of exposure to federal court–ordered antismoking advertisements using a nationally representative sample of US adults.

## Methods

### Study Population, Design, and Setting

Data for this study were obtained from the 2018 Health Information National Trends Survey (HINTS) 5, Cycle 2, a nationally representative survey of US adults 18 years or older that was administered by the National Cancer Institute. The sample frame for HINTS 5, Cycle 2 was derived using the Marketing Systems Group database of addresses and included all nonvacant residential addresses in the United States. In this sampling frame, addresses were grouped into low- and high-minority strata. The high-minority strata was oversampled to enhance accuracy of estimates for this population. An equal-probability method was used to select addresses within each stratum, and then 1 adult per sampled household was selected to participate in the survey. The overall response rate for HINTS 5, Cycle 2 was 32.9% using the Response Rate 2 formula of the American Association of Public Opinion Research^12^ and was comparable to previous cycles of HINTS.^13^ Written informed consent was obtained from study participants. The Westat institutional review board approved HINTS 5, Cycle 2, and it was classified exempt from review by the US National Institutes of Health Office of Human Subjects Research Protections. This report follows the American Association of Public Opinion Research (AAPOR) reporting guideline. A detailed description of survey methodology has been published.^14^

The US population that participated in the survey was exposed to the antismoking advertisements from November 2017 to May 2018. Television advertisements ran from November 2017 to November 2018, and newspaper advertisements ran from November 2017 to May 2018. The HINTS 5, Cycle 2 data were collected from January 26, 2018, to May 2, 2018.

Survey questionnaire responses were returned by mail and were documented by month and period of return. Frequencies by months of return are as follows: February (n = 2269), March (n = 877), and April/May (n = 338). April and May were merged because all surveys were returned by May 2.

### Study Variables

#### Outcome Measure

The main outcome measure of this study was self-reported exposure to a court-ordered antismoking advertisement. It was defined as a yes response to the question, “In the past 6 months, have you seen messages in newspapers or on television that say that a federal court has ordered tobacco companies to make statements about the dangers of smoking cigarettes?”

#### Participant Characteristics

Several factors were considered in the evaluation of the reach of the court-ordered antismoking advertisements, including age, sex, race/ethnicity, level of education, rural-urban residence, household annual income, and smoking status. Age was grouped into 4 categories as follows: 18 to 34 years, 35 to 49 years, 50 to 64 years, and 65 years or older. Race/ethnicity was categorized as non-Hispanic white, non-Hispanic black, and Hispanic. Level of education was grouped into 3 categories: high school graduate or less, post–high school or some college (a combination of post–high school training other than college and some college training), and college graduate or postgraduate. Residence was defined using the US Department of Agriculture’s 2003 Rural-Urban Continuum Codes. Codes 1 to 3 were designated as urban, while codes 4 to 9 were categorized as rural. Household annual income was categorized as less than $35 000, $35 000 to $49 999, $50 000 to $74 999, and $75 000 or more. To derive respondents’ smoking status, respondents were asked the question, “Have you smoked at least 100 cigarettes in your entire life?” Those who answered no were categorized never smokers. Among those who answered yes, a follow-up question was asked: “Do you now smoke cigarettes every day, some days, or not at all?” Those who answered not at all were categorized former smokers, while others were considered current smokers.

#### Type of Court-Ordered Antismoking Advertisement Seen

Respondents who reported exposure to court-ordered antismoking messages were asked a follow-up question: “Which of the following messages about the dangers of smoking cigarettes have you seen?” Possible responses were: (1) “federal court–ordered tobacco messages: health effects of smoking”; (2) “federal court–ordered tobacco messages: health effects of secondhand smoke”; (3) “federal court–ordered tobacco messages: addictiveness”; (4) “federal court–ordered tobacco messages: enhanced delivery”; (5) “federal court–ordered tobacco message: low-tar and light cigarettes”; and (6) “multiple federal court–ordered tobacco messages.”

### Statistical Analysis

Prevalence of exposure to the court-ordered antismoking messages were estimated for the overall sample as well as by age, sex, race/ethnicity, level of education, rural-urban residence, household annual income, and tobacco use characteristics. To evaluate the association of the duration of the advertising campaign with exposure prevalence, antismoking advertisement exposure was estimated by the month survey responses were returned by mail. To do this, respondents’ exposure status was paired to the month when the survey questionnaire was returned. We considered the duration of exposure to begin in November 2017, when the antismoking advertisements commenced, and end the month the survey was returned.

Because smokers have a heightened tendency to be attuned to smoking-related advertisements, we examined exposure prevalence by sociodemographic characteristics among a subpopulation of current smokers. The type of antismoking message that participants reported exposure to was evaluated for the general population and for subgroups stratified by smoking status. Factors associated with exposure were explored using multivariable survey logistic regression. Statistical significance was defined as a *P* value less than .05, and all tests were 2-tailed. All data were weighted to be nationally representative and analyzed with SAS version 9.4 (SAS Institute).

## Results

The overall sample of 3484 respondents included 2054 women (weighted percentage, 50.8%), 1976 non-Hispanic white respondents (weighted percentage, 59.9%), 2952 respondents who lived in urban US areas (weighted percentage, 84.9%), and 450 current smokers (15.6%). In the full sample, estimated exposure to US Federal Court–ordered antismoking advertisements was 40.6% (95% CI, 37.5%-43.7%) (Table 1). Exposure was lowest among those aged 18 to 34 years (37.4%; 95% CI, 28.0%-46.8%), those with a high school education or less (34.5%; 95% CI, 29.3%-39.8%), and those with a household annual income less than $35 000 (37.5%; 95% CI, 32.0%-42.9%).

**Table 1.** Exposure to US Federal Court–Ordered Antismoking Advertisements by Sociodemographic Characteristics and Smoking Status

Characteristic	Respondents With Exposure		Respondents by Period of Exposure^a^					
			November 2017 to February 2018		November 2017 to March 2018		November 2017 to April/May 2018	
	No.	% (95% CI)^b^	No.	% (95% CI)^b^	No.	% (95% CI)^b^	No.	% (95% CI)^b^
Overall	3484	40.6 (37.5-43.7)	2269	41.3 (37.9-44.6)	877	37.2 (30.4-44.1)	338	46.8 (35.5-58.1)
Sex								
Male	1394	42.6 (37.1-48.0)	901	42.8 (36.2-49.4)	372	40.1 (29.9-50.3)	121	49.3 (31.3-67.3)
Female	2054	39.0 (36.0-41.9)	1349	39.8 (35.8-43.7)	490	35.0 (28.4-41.5)	215	44.7 (32.8-56.6)
Age, y								
18-34	406	37.4 (28.0-46.8)	239	37.8 (26.0-49.5)	118	34.2 (14.2-54.2)	49	46.4 (23.1-69.8)
35-49	655	41.0 (34.4-47.5)	399	40.9 (33.3-48.4)	177	41.3 (30.6-51.9)	79	40.6 (20.9-60.3)
50-64	1108	42.8 (37.4-48.3)	711	44.0 (36.9-51.0)	298	36.5 (29.6-43.4)	99	55.6 (37.5-73.7)
≥65	1237	40.4 (36.2-44.6)	869	41.7 (36.5-46.9)	268	34.9 (25.5-44.2)	100	45.2 (31.6-58.8)
Race/ethnicity								
Non-Hispanic white	1976	40.7 (36.5-44.8)	1364	40.8 (36.6-45.0)	455	37.5 (27.6-47.4)	157	50.0 (33.0-66.9)
Non-Hispanic black	444	41.5 (34.5-48.6)	276	39.2 (29.7-48.7)	117	39.3 (27.5-51.0)	51	63.6 (47.9-79.3)
Hispanic	460	42.2 (33.7-50.8)	252	48.7 (39.0-58.4)	143	35.3 (21.4-49.1)	65	35.9 (17.0-54.8)
Level of education								
College graduate or postgraduate	1506	41.6 (38.4-44.8)	1001	40.9 (36.7-45.1)	363	41.5 (34.7-48.3)	142	46.5 (32.7-60.3)
Post–high school or some college	1036	44.5 (38.6-50.4)	676	44.1 (37.5-50.6)	261	43.9 (29.9-57.9)	99	49.2 (29.6-68.9)
≤High school graduate	902	34.5 (29.3-39.8)	562	36.9 (31.1-42.7)	244	27.5 (16.6-38.5)	96	44.5 (27.0-61.9)
Residence								
Urban	2952	40.5 (37.2-43.7)	1899	41.0 (37.1-44.8)	764	37.8 (30.4-45.2)	289	45.6 (33.8-57.5)
Rural	532	41.5 (34.9-48.1)	370	42.9 (35.7-50.2)	113	33.6 (19.0-48.2)	49	54.3 (30.5-78.2)
Household annual income, $								
<35 000	1000	37.5 (32.0-42.9)	619	36.7 (29.9-43.5)	271	33.7 (22.6-44.9)	110	52.2 (29.2-75.2)
35 000-49 999	404	39.4 (31.0-47.8)	271	44.7 (35.7-53.8)	96	30.0 (11.5-48.5)	37	35.2 (11.4-59.0)
50 000-74 999	566	39.1 (30.5-47.6)	392	37.2 (28.8-45.6)	130	40.7 (13.3-68.0)	44	48.7 (22.1-75.2)
≥75 000	1108	44.5 (40.3-48.7)	731	46.0 (40.1-52.0)	288	39.5 (31.4-47.6)	89	51.2 (37.1-65.3)
Smoking status								
Never	2129	38.6 (34.4-42.9)	1359	40.9 (36.9-44.9)	561	34.2 (24.5-44.0)	209	38.9 (25.5-52.2)
Former	865	40.7 (34.9-46.4)	587	40.5 (33.8-47.1)	201	38.8 (27.6-50.1)	77	48.2 (32.5-63.9)
Current	450	50.5 (41.4-59.6)	291	46.6 (36.2-57.1)	111	48.9 (30.5-67.3)	48	78.3 (64.3-92.2)

^a^ Duration of advertisement coverage at time of survey submission.

^b^ Results represent the number and weighted percentage of respondents who replied yes to the following question: “In the past 6 months, have you seen messages in newspapers or on television that say that a federal court has ordered tobacco companies to make statements about the dangers of smoking cigarettes?”

Analysis of antismoking advertisement exposure by duration of advertising campaign revealed that as the advertising campaign’s duration increased, so did rates of reported exposure (Table 1). Individuals who returned surveys in April/May (at which time advertisements had run for 6 to 7 months) had the highest exposure rates in the overall population (46.8%; 95% CI, 35.5%-58.1%). This observation was most pronounced among current smokers, with exposure increasing from 46.6% (95% CI, 36.2%-57.1%) for surveys returned in February 2018 to 48.9% (95% CI, 30.5%-67.3%) for those returned in March 2018 and 78.3% (95% CI, 64.3%-92.2%) for surveys returned in April/May 2018.

Among the subpopulation of current smokers (Table 2), we found that 50.5% (95% CI, 41.4%-59.6%) reported seeing court-ordered antismoking messages. Corresponding with findings in the general population, exposure rates among current smokers were lowest among those aged 18 to 34 years (45.2%; 95% CI, 24.1%-66.4%). However, unlike the findings in the general population, exposure rates among current smokers were lower among Hispanic respondents (42.2%; 95% CI, 18.5%-65.9%) than non-Hispanic white respondents (51.7%; 95% CI, 40.4%-63.1%).

**Table 2.** Exposure to US Federal Court–Ordered Antismoking Advertisements by Sociodemographic Characteristics Among 450 Current Smokers

Characteristic	Total Respondents, No.	No^a^		Yes^a^	
		No.	% (95% CI)	No.	% (95% CI)
Overall	450	211	49.5 (40.4-58.6)	239	50.5 (41.4-59.6)
Sex					
Male	205	109	54.8 (42.7-66.9)	96	45.2 (33.1-57.3)
Female	241	102	42.0 (31.3-52.7)	139	58.0 (47.3-68.7)
Age, y					
18-34	47	24	54.8 (33.6-75.9)	23	45.2 (24.1-66.4)
35-49	103	45	49.1 (28.6-69.7)	58	50.9 (30.3-71.4)
50-64	189	90	47.9 (33.2-62.7)	99	52.1 (37.3-66.8)
≥65	110	51	47.0 (34.6-59.3)	59	53.0 (40.7-65.4)
Race/ethnicity					
Non-Hispanic white	240	109	48.3 (36.9-59.6)	131	51.7 (40.4-63.1)
Non-Hispanic black	70	29	50.7 (17.9-83.5)	41	49.3 (16.5-82.1)
Hispanic	53	30	57.8 (34.1-81.5)	23	42.2 (18.5-65.9)
Level of education					
College graduate or postgraduate	101	45	51.4 (36.5-66.3)	56	48.6 (33.7-63.5)
Post–high school or some college	179	84	46.4 (34.8-58.1)	95	53.6 (41.9-65.2)
≤High school graduate	170	82	51.6 (35.4-67.9)	88	48.4 (32.1-64.6)
Residence					
Urban	377	181	50.6 (41.0-60.2)	196	49.4 (39.8-59.0)
Rural	73	30	43.4 (25.0-61.8)	43	56.6 (38.2-75.0)
Household annual income, $					
<35 000	202	97	47.6 (34.8-60.5)	105	52.4 (39.5-65.2)
35 000-49 999	61	27	43.3 (17.3-69.2)	34	56.7 (30.8-82.7)
50 000-74 999	61	35	64.1 (49.1-79.1)	26	35.9 (20.9-50.9)
≥75 000	88	34	50.7 (28.3-73.2)	54	49.3 (26.8-71.7)

^a^ Response to the following question: “In the past 6 months, have you seen messages in newspapers or on television that say that a federal court has ordered tobacco companies to make statements about the dangers of smoking cigarettes?”

Among those who reported exposure to court-ordered antismoking messages, the Figure shows the type of antismoking messages seen, stratified by smoking status. Overall, 70.5% of respondents reported seeing multiple antismoking messages; this population included 79.0% of current smokers, 65.2% of former smokers, and 69.4% of never smokers. Of the individual message categories, messages on the health effects of smoking were the most frequently reported (17.3%); the population that reported seeing these messages included 12.1% of current smokers, 16.1% of former smokers, and 19.4% of never smokers. Advertisements regarding the design of tobacco products for enhanced nicotine delivery were the least reported.

**Figure.**
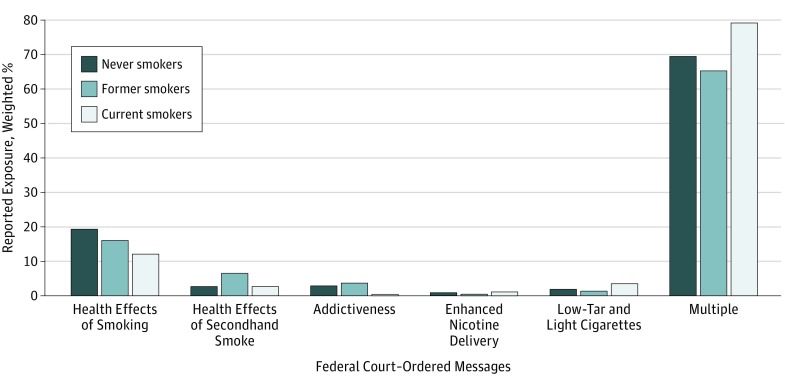
Weighted Percentage of Reported Exposure to Federal Court–Ordered Messages Among US Adults by Smoking Status

Our multivariable logistic regression analysis revealed that, following adjustment, exposure odds were lower among those who had a level of education of high school or less compared with those who had a college or postgraduate degree (adjusted odds ratio, 0.67; 95% CI, 0.48-0.94) (Table 3). The odds of exposure to antismoking advertisements were higher among current smokers than never smokers (adjusted odds ratio, 1.81; 95% CI, 1.17-2.80) (Table 3).

**Table 3.** Adjusted Multivariable Logistic Regression of Exposure to US Federal Court–Ordered Antismoking Advertisements by Sociodemographic Characteristics and Smoking Status

Characteristic	Adjusted Odds Ratio (95% CI)	*P* Value
Sex		
Male	1 [Reference]	NA
Female	0.90 (0.69-1.17)	.43
Age, y		
18-34	1 [Reference]	NA
35-49	1.03 (0.65-1.64)	.91
50-64	1.19 (0.75-1.88)	.46
≥65	1.23 (0.78-1.94)	.37
Race/ethnicity		
Non-Hispanic white	1 [Reference]	NA
Non-Hispanic black	1.15 (0.75-1.76)	.51
Hispanic	1.26 (0.83-1.91)	.26
Level of education		
College graduate or postgraduate	1 [Reference]	NA
Post–high school or some college	1.11 (0.80-1.54)	.53
≤High school graduate	0.67 (0.48-0.94)	.02
Residence		
Urban	1 [Reference]	NA
Rural	1.14 (0.86-1.50)	.36
Household annual income, $		
<35 000	1 [Reference]	NA
35 000-49 999	1.09 (0.74-1.59)	.66
50 000-74 999	1.03 (0.65-1.64)	.89
≥75 000	1.30 (0.96-1.78)	.09
Smoking status		
Never	1 [Reference]	NA
Former	1.05 (0.75-1.48)	.78
Current	1.81 (1.17-2.80)	.009

Abbreviation: NA, not applicable.

## Discussion

To our knowledge, this study is the first to examine the reach of the federal court–ordered antismoking advertising campaign sponsored by the tobacco industry within the US population. In a nationally representative sample of US adults, the estimated prevalence of antismoking advertisement exposure was 40.6% among the overall study population and 50.5% among current smokers.

Compared with the exposure rates of antismoking campaigns conducted by federal and state public health agencies, penetration of the tobacco industry–sponsored antismoking advertisements was suboptimal. The first federally funded antismoking campaign called Tips from Former Smokers, administered by the US Centers for Disease Control and Prevention, achieved an exposure rate of almost 80% among smokers despite running for a shorter duration (3 months) than the industry-sponsored advertising campaign.^6^ Similarly, studies of state-level antitobacco advertising campaigns in Massachusetts^15^ and California^16^ recorded 88% and 69.5% exposure rates, respectively, within their overall study cohorts.

Of additional concern was our finding that the youngest members of the study sample, those in the lowest income bracket, and those with the lowest level of education registered the lowest rates of exposure to the court-ordered antismoking advertisements. In our subset analysis of current smokers, the lowest exposure rates were observed in the youngest members (consistent with the overall sample), while Hispanic respondents were found to have lower exposure rates than non-Hispanic respondents. Given the tobacco industry’s history of aggressively targeting young people as well as racial minorities and socially disadvantaged (eg, poorly educated) populations,^17,18,19^ it was critical that the corrective statements reach these populations.

Our study also found that, as the advertising campaign’s duration lengthened, exposure rates improved significantly in the general population and in all demographic groups. This finding lends support to the recommendations of studies (conducted before and after finalization of the corrective statements)^10,20^ that call for repeated exposure as a means to ensure a sustained effect.

While these federal court–ordered advertising campaigns represent a critical point in tobacco control from a historical and legal perspective, our findings suggest that their real-world association with preventing initiation and promoting cessation may be limited on account of their suboptimal population-level penetrance, especially among young adults, who are at greatest risk for smoking initiation. Careful consideration of medium and communication style for these advertisements is important to increase their effect; for example, placement in youth-oriented channels, including social media, may help increase awareness among young people.^21,22^ Furthermore, increasing the dose of advertising has proven potential to boost population-level exposure during antismoking campaigns.^23^

Findings from this study could potentially inform effective implementation and evaluation of ongoing court-ordered placement of corrective statements on cigarette package onserts and tobacco company websites as well as anticipated antismoking advertising at point-of-sale displays (the subject of ongoing litigation).^5^ Epidemiologic studies would also be needed to evaluate the effect of these advertisements on smoking-related knowledge, intentions, and behaviors, including initiation, cessation, and tobacco consumption patterns overall.

The US federal court ruling on corrective statements raises questions on what constitutes deliberate deceptive practices that need correcting. It could be argued that such deceptive behavior is not limited to text or verbal statements made by representatives of the industry in the past but also includes current and ongoing deception in the form of glamorous or attractive tobacco product design and marketing that implies reduced harm or some healthful benefits. This implication of reduced harm is also a health equity issue given the marketing of several products has been targeted at minorities (eg, menthol cigarettes), which has resulted in disparities in tobacco use and tobacco-related morbidity and mortality.

### Limitations

Despite the strengths of this study, there are a number of limitations. First, HINTS data are cross-sectional, and hence causal inferences cannot be made. Second, HINTS data are self-reported and prone to recall and social desirability bias. Further, we could not independently assess exposure by advertising medium (television or newspaper) because these data were pooled.

## Conclusions

This study found that the unprecedented national-level antismoking advertising campaign sponsored by the tobacco industry had a suboptimal reach within the US population. Penetration rates were even lower in known at-risk groups, such as young adults. This study offers critical cues for oversight of ongoing and future tobacco industry–sponsored antismoking advertising campaigns. This study also draws attention to the important role that the judiciary, along with regulatory agencies, public health agencies, and health interest groups, can play in comprehensive tobacco control and prevention. District Judge Kessler’s landmark ruling could have implications for global tobacco control (if similar efforts are adopted by the judiciary in regions outside the United States) as well as beyond the field of tobacco control and may set a precedent for similar actions in other areas relevant to public health where deceptive industry marketing practices exist.
